# First do no harm: An exploration of researchers’ ethics of conduct in Big Data behavioral studies

**DOI:** 10.1371/journal.pone.0241865

**Published:** 2020-11-05

**Authors:** Maddalena Favaretto, Eva De Clercq, Jens Gaab, Bernice Simone Elger

**Affiliations:** 1 Institute for Biomedical Ethics, University of Basel, Basel, Switzerland; 2 Division of Clinical Psychology and Psychotherapy, Faculty of Psychology, University of Basel, Basel, Switzerland; Rowan University School of Osteopathic Medicine, UNITED STATES

## Abstract

Research ethics has traditionally been guided by well-established documents such as the Belmont Report and the Declaration of Helsinki. At the same time, the introduction of Big Data methods, that is having a great impact in behavioral research, is raising complex ethical issues that make protection of research participants an increasingly difficult challenge. By conducting 39 semi-structured interviews with academic scholars in both Switzerland and United States, our research aims at exploring the code of ethics and research practices of academic scholars involved in Big Data studies in the fields of psychology and sociology to understand if the principles set by the Belmont Report are still considered relevant in Big Data research. Our study shows how scholars generally find traditional principles to be a suitable guide to perform ethical data research but, at the same time, they recognized and elaborated on the challenges embedded in their practical application. In addition, due to the growing introduction of new actors in scholarly research, such as data holders and owners, it was also questioned whether responsibility to protect research participants should fall solely on investigators. In order to appropriately address ethics issues in Big Data research projects, education in ethics, exchange and dialogue between research teams and scholars from different disciplines should be enhanced. In addition, models of consultancy and shared responsibility between investigators, data owners and review boards should be implemented in order to ensure better protection of research participants.

## Introduction

Big Data methods have a great impact in behavioral sciences [1–3], but challenge the traditional interpretation and validity of research principles in psychology and sociology by raising new and unpredictable ethical concerns. Traditionally, research ethics have been guided by well-established reports and declarations such as the Belmont Report and the Declaration of Helsinki [4–6]. At the core of these documents are three fundamental principles–*respect for persons*, *beneficence*, and *justice*–and their related interpretations and practices, such as the acknowledgment of participants’ autonomous participation and the need to obtain informed consent, minimization of harm, risk benefit assessment, fairness in distribution and dissemination of research outcomes, and fair participant selection (e.g. to avoid additional burden to vulnerable populations) [7].

As data stemming from human interactions is more and more available to scholars, thanks to a) the increased distribution of technological devices, b) the growing use of digital services, and c) the implementation of new digital technologies [8, 9], researchers and institutional bodies are confronted with novel ethical questions. These encompass harm, that might be caused by the linkage of publicly available datasets on research participants [10], the level of privacy users expect in digital platforms such as social media [11], the level of protection that investigators should ensure for the anonymity of their participants in research using sensing devices and tracking technologies [12], and the role of individuals in consenting in participating in large scale data studies [13].

Consent is one of the most challenged practices in data research. In this context subjects are often unaware of the fact that their data is collected and analyzed and lack the appropriate control over their data, preventing them the possibility to withdraw from a study, that allows for autonomous participation [14, 15]. When it comes to the principle of *beneficence*, Big Data brings about issues with regard to the appropriate risk-benefit ratio for participants as it becomes more difficult for researchers to anticipate unintended harmful consequences [8]. For example, it is increasingly complicated to ensure anonymity of the participant as risks of re-identification abound in Big Data practices [12]. Finally, interventions and knowledge developed from Big Data research might benefit only part of the population thus creating issues of *justice* and fairness [10]; this is mainly due to the deepening of the digital divide between people who have access to digital resources and those who do not, on the basis of a significant number of demographic variables such as income, ethnicity, age, skills, geographical location and gender [10, 16].

There is evidence that researchers and regulatory bodies are struggling to appropriately address these novel ethical questions raised by Big Data. For instance, a group of researchers based at Queen’s Mary University in the UK used a model of geographic profiling on a series of publicly available datasets in order to reveal the identity of famous British artist Banksy [17]. The study was criticized by scholars for being disrespectful of the privacy of a private citizen and their family and a deliberate violation of the artist’s right of and preference for remaining anonymous [18]. Another example is the now infamous case of the Emotional Contagion study. Using a specific software, a research team manipulated the News Feeds of 689,003 Facebook users in order investigate how “emotional states can be transferred to others via emotional contagion, leading people to experience the same emotions without their awareness” [19]. Ethics scholars and the public criticized this study because it was performed without obtaining the appropriate consent from Facebook users and it could have cause psychological harm by showing participants only negative feeds on their homepage [20, 21].

Given these substantial challenges, it is legitimate to ask whether the principles set by the Belmont Report are still relevant for digital research practices. Scholars advocate for the construction of flexible guidelines and for the need to revise, reshape and update the guiding principles of research ethics in order to overcome the challenges raised in data research and provide adequate assistance to investigators [22–24].

As ethics governance of Big Data research is currently at debate, researchers’ own ethical attitudes influence significantly how ethical issues are presently dealt with. As researchers are experts on the technical details of their own research, it is also useful for research ethicists and members of ethical committees and Institutional Review Boards (IRB) to be knowledgeable of these attitudes. Therefore, this paper aims to explore the code of ethics and research practices of behavioral scientists involved in Big Data studies in the behavioral sciences in order to investigate perceived strategies to promote ethical and responsible conduct of Big Data research. We have conducted interviews with researchers in the fields of sociology and psychology from eminent universities both in Switzerland and the United States, where we asked them to share details about the type of strategies they develop to protect research participants in their projects; what ethical principles they apply to their projects; their opinion on how Big Data research should ideally be conducted and what ethical challenges they have faced in their research. The present study aims to contribute to the existing literature on the code of conduct of researchers involved in digital research in different countries and the value of traditional ethical principles [14, 22, 23] in order to contribute to the discussion around the construction of harmonized and applicable principles for Big Data studies. This manuscript aims at investigating the following research questions: 1) what are the ethical principles that can still be considered relevant for Big Data research in the behavioral sciences; 2) what are the challenges that Big data methods are posing to traditional ethical principles; 3) what are the investigators’ responsibilities and roles in reflecting upon strategies to protect research participants.

## Material and methods

This study is part of a larger research project that investigated the ethical and regulatory challenges of Big Data research. We decided to focus on behavioral sciences, specifically phycology and sociology, for two main reasons. First, the larger research project aimed at investigating the challenges introduced by Big Data methods for regulatory bodies such as Research Ethics Committees (RECs) and Institutional Review Boards (IRBs) [25]. Both in Switzerland and the United States, Big Data research methods in these two fields are questioning the concept of human research subject–due to the increased distance and detachment between research subjects and investigators brought by digitalized means for data collection (e.g. social media profiles, data networks, transaction logs etc.) and analysis [18]. As a consequence current legislation in charge of regulating academic research, such as the Human Research Act (HRA) [26], the Federal Act of Data Protection [27] and the Common Rule [18], is being increasingly challenged. Second, especially in Switzerland, behavioral studies using Big Data methods are at the moment among the most underregulated types of research projects [26, 28, 29]. In fact, the current definition of human subject leaves many Big Data projects out of the scope of regulatory overview despite the possible ethical challenges they pose. For instance, according to the HRA research that involves anonymized data from research participants does not need ethics approval [26].

In addition, we selected Switzerland and the United States to recruit participants: Switzerland, where Big Data research is a quite recent phenomenon, was chosen because the study was designed, funded and conducted there. The United States were selected as a as a comparative sample, where advanced Big Data research has been taking place for several years in the academic environment, as evidenced by the numerous grants placed for Big Data research projects by federal institutions, such as the National Science Foundation (NSF) [30, 31] and the National Institute of Health (NIH) [32].

For the purpose of our study we defined Big Data as an overarching umbrella term that designates a set of advanced digital techniques (e.g. data mining, neural networks, deep learning, artificial intelligence, natural language processing, profiling, scoring systems) that are increasingly used in research to analyze large datasets with the aim of revealing patterns, trends and associations about individuals, groups and society in general [33]. Within this definition we selected participants that conducted heterogeneous Big Data research projects: from internet-based research and social media studies, to aggregate analysis of corporate datasets, to behavioral research using sensing devices. Participant selection was based on their involvement in Big Data research and was conducted systematically by browsing the professional pages of all professors affiliated to the departments of psychology and sociology of all twelve Swiss Universities and the top ten American Universities according to the Times Higher Education University Ranking 2018. Other candidates were identified through snowballing. Through our systematic selection we also identified a consistent number of researchers with a background in data science that were involved in research projects in behavioral sciences (in sociology, psychology and similar fields) during the time of their interview. Since their profile matched the selection criteria, we included them in our sample.

We conducted 39 semi structured interviews with academic scholars involved in research projects that adopt Big Data methodologies. Twenty participants were from Swiss universities and 29 came from American institutions. They comprised of a majority of professors (n = 34) and a few senior researchers or postdocs (n = 5). Ethics approval was sought from the Ethics Committee northwest/central Switzerland (EKNZ) who deemed our study exempt. Oral informed consent was sought prior the start of each interview. Interviews were administered using a semi-structured interview guide developed, through consensus and discussion, after the research team had the time to familiarize with the literature and studies on Big Data research and data ethics. The questions explored topics like: ethical issues related to Big Data studies in the behavioral sciences; ethics of conduct with regards to Big Data research project; institutional regulatory practices; definition and understanding of the term Big Data; and opinions towards data driven studies (Table 1).

**Table 1:** Relevant interview questions.

Sample questions
Was it clear to you which kind of ethical guidelines you would have to apply to your research? Are there any specific guidelines that you applied to conduct your research?
Do you find the guidelines that you are currently using useful? Anything that bothers you about them? Do you have any suggestion on how to improve them?
How do you think data research should be ideally ethically regulated?
What are in your opinion the minimal requirements that the law should enact to ensure that data research is carried out with minimal challenges but fulfilling ethical requirements?
What do you think is the main difference between Big Data research and more conventional research in your field? Do you think this has any implications for the guidelines?
Have you encountered any particular (ethical) challenges when conducting your research project?

Interviews were tape recorded and transcribed ad-verbatim. We subsequently transferred the transcripts into the qualitative software MAXQDA (version 2018) to support with data management and the analytic process [34]. Analysis of the dataset was done using thematic analysis [35]. The first four interviews were independently read and coded by two members of the research team in order to explore the thematic elements of the interviews. To ensure consistency during the analysis process, the two researchers subsequently confronted the preliminary open-ended coding and they developed an expanded coding scheme that was used for all of the remaining transcripts. Several themes relevant for this study were agreed upon during the coding sessions such as: a) responsibility and the role of the researcher in Big Data research; b) research standards for Big Data studies; c) attitudes towards the use of publicly available data; d) emerging ethical issues from Big Data studies. Since part of the data has already been published, we refer to a previous publication [33] for additional information on methodology, project design, data collection and data analysis.

Researcher’s code of ethics for Big Data studies was chosen as a topic to explore since participants, by identifying several ethical challenges related to Big Data, expressed concerns regarding the protection of the human subject in digital research and expressed shared strategies and opinions on how to ethically conduct Big Data studies. Consequently, all the interviews that were coded within the aforementioned topics were read again, analyzed and sorted into sub-topics. This phase was performed by the first author while the second author supervised this phase by checking for consistency and accuracy.

## Results

For this study we conducted 39 interviews with respectively 21 sociologists (9 from CH and 12 from the US), 11 psychologists (6 from CH and 5 from the US), and 7 data scientists (5 from CH and 2 from the US). Among them, 27 scholars (12 from CH and 21 from US) stated that they were working on Big Data research projects or on projects that involve Big Data methodologies, four participants (all from CH) noted that they were not involved in Big Data research and eight (7 from CH and one from the US) were unsure whether their research could be described or considered as Big Data research (Table 2).

**Table 2:** Demographic table.

	Psychology (P)	Sociology (S)	Data Science (D)	Total
**CH Researchers**	6	9	5	20
**US Researchers**	5	12	2	19
**Professors**	9	20	5	34
**Postdocs/Senior researchers**	2	1	2	5

Respondents, while discussing codes of ethics and ethical practices for Big Data research, both a) shared their personal strategies that they implemented in their own research projects to protect research subjects, and b) generally discussed the appropriate research practices to be implemented in Big Data research. Table 3 illustrates the type of Big Data our participants were working with at the time of the interview.

**Table 3:** Type of data used by participants.

Type of data	Participant Number*
Data From Companies (anonymized/aggregate purchase data, traffic phone data)	P29CH-D; P35CH-S; P38CH-S; P1US-S; P18US-D.
Sensing Devices and Sensor data (smartphone data, GPS, fitness trackers, Wi-Fi interactions)	P22CH-P; P28CH-S; P38CH-S; P4US-P; P18US-D; P20US-S; P22US-S.
Social Media Data (Twitter, Facebook, GAAB, Telegram, Reddit)	P24CH-P; P28CH-S; P29CH-D; P3US-S; P12US-S; P18US-D; P20US-S; P21US-S; P22US-S.
Physiological Data (EG, eye tracking)	P22CH-P; P8US-D; P22US-S.
Medical Data (neuroimaging, blood samples, x-rays, genetic data)	P1CH-P; P31CH-D; P32CH-D; P34CH_D; P4US-P; P9US-S; P11US-P; P12US-S; P13US-P; P14US-P; P16US-S.
Administrative data (university and state records, federal records, juridical, tax and census data)	P33CH-S; P39CH-S; P4US-P; P6US-S.
Publicly available data (newspaper, books, websites, public documents, data on public figures)	P23CH-S; P30CH-S; P35CH-S; P37CH-S; P1US-S; P2US-S; P3US-S; P6US-S; P17US-P; P19US-S; P20US-S.
Interview and Survey Data	P24CH-P; P28CH-S; P29CH-D; P39CH-S; P2US-S; P4US-P; P14US-P; P17US-P.
Crowdsourcing Data (M-Turk, Crowd Flower, Safecast)	P27CH-D; P29CH-S; P20US-S.
Not specified	P5US-S.

*: P = participant+ID number+country (CH = Switzerland; US = United States)+background (P = Psychology; S = Sociology; D = Data Science). Eg. P1CH-P = Participant 1, Switzerland, Psychology.

Our analysis identified several themes and subthemes. They were then divided and analyzed within three major thematic clusters: a) ethical principles for Big Data research; b) challenges that Big Data is introducing for research principles; c) ethical reflection and responsibility in research. Table 4 reports the themes and subthemes that emerged from the interviews and their occurrence in the dataset. Representative anonymized quotes were taken from the interviews to further illustrate the reported results.

**Table 4:** Themes and clusters that emerged from the analysis.

Themes and subthemes	Number of occurrences in the dataset*	Cluster 1: ethical principles for Big Data research	Cluster 2: challenges for research principles	Cluster 3: ethical reflection and responsibility in research
1. Responsibility	16			x
1.1 Responsibility to protect the research subject lies on the investigators primarily	10			x
1.2 Investigators cannot be the only actors held responsible or Big Data research	6			x
2. Role and importance of ethical reflection and ethical principles	5			x
3. Research Guidelines	3	x		
3.1 Belmont Report	2	x		
3.2 Declaration of Helsinki	1	x		
4. Research Principles	99	x	x	
4.1 Beneficence	5	x		
4.2 Avoiding Harm	4	x		
4.3 Respect for the participant	2	x		
4.4 Consent	40	x	x	
4.4.1 Importance of consent	19	x		
4.4.2 Awareness of participants	4	x		
4.4.3 Consent is challenging in Big Data research	14		x	
4.4.4 Consent is not the most relevant research principle	3		x	
4.5 Right to withdraw and control over one's data	5	x		
4.6 Privacy	34	x	x	
4.6.1 Importance of respecting people's privacy in research	10	x		
4.6.2 Ensuring participants' anonymity	6	x		
4.6.3 Big Data is challenging the concept of privacy	7		x	
4.6.3.1 The public versus private data conundrum	11		x	
4.7 Transparency	9	x		
4.7.1 Clash between transparency and anonymity	1		x	
4.7.2 Importance of evaluation of intent	1	x		x

* By occurrence we refer to the number of times a theme or a subtheme was coded within the data. It is therefore possible that a single participant mentioned the same concept/topic more than one time during the interview. In addition, a single quote could refer to more than one theme.

### Ethical principles for digital research

#### Belmont principles, beneficence and avoiding harm

First, many of the respondents shared their opinions on what ethical guidelines and principles they consider important to conduct ethical research in the digital era. Table 5 illustrates the number of researchers that mentioned a specific ethical principle or research practice as relevant for Big Data research.

**Table 5:** Mentioned ethical principles.

Research Principles and Practices	Swiss Scholars	American Scholars	Total
**Belmont Report/Declaration of Helsinki**	1	2	3
**Avoiding Harm**	1	2	3
**Beneficence/Giving Back to the Community**	1	3	4
**Respect**	1	1	2
**Informed Consent**	9	10	19
**Awareness**	2	2	4
**Right to withdraw/ Control over data**	2	2	4
**Transparency**	5	4	9
**Privacy/Anonymity**	8	7	15
**Evaluation of intent**	1	0	1

Three of our participants, generally referred to the principles stated in the Belmont Report and the ones related to the Declaration of Helsinki.

I think the Belmont Report principles. The starting point so. . . .you know beneficence, respect for the individuals, justice… and applying those and they would take some work for how to apply those exactly or what it would mean translating to this context but that would be the starting point (P18, US–data science).

A common concern was minimization of harm for research participants and the importance of beneficence as prominent components of scholarly research.

And…on an ethical point of view… and I guess we should be careful that experiment doesn’t harm people or not offend people for example if it's about religion or something like that it can be tricky (P25, CH–psychology).

Beneficence, in the context of digital Big Data research, was sometimes associated with the possibility of giving back to the community as a sort of tradeoff for the inconvenience that research might cause to research participants. On this, P9, an American sociologist, shared:

I mean it's interesting that the ethical challenges that I faced… (pause) had more to do with whether I feel, for instance in working in the developing world…is it really beneficial to the people that I'm working with, I mean what I'm doing. You know I make heavy demands on these people so one of the ethical challenges that I face is, am I giving back enough to the community.

While another American scholar, a psychologist, was concerned about how to define acceptable risks in digital research and finding the right balance between benefit and risks for research projects.

P17: Expecting benefit from a study that should outweigh the respective risks. I mean, I think that's a pretty clear one. This is something I definitely I don't know the answer to and I'm curious about how much other people have thought about it. Because like what is an acceptable sort of variation in expected benefits and risks. Like, you could potentially say “on average my study is expected to deliver higher benefits than risks”… there's an open question of like, … some individuals might regardless suffer under your research or be hurt. Even if some others are benefitting in some sense.

For two researchers, respect for the participant and their personhood was deemed particularly important irrespective of the type of research conducted. P19, an American sociologist, commented:

What I would like to see is integrity and personhood of every single individual who is researched, whether they are dead or alive, that that be respected in a very fundamental way. And that is the case whether it's Big Data, and whether is interviews, archival, ethnographic, textual or what have you. And I think this is a permanent really deep tension in wissenshaftlich (*scientific research*) activities because we are treating the people as data. And that's a fundamental tension. And I think it would be deeply important to explicitly sanitize that tension from the get-go and to hang on to that personhood and the respect for that personhood.

#### Informed consent and transparency

Consent was by far the most prominent practice that emerged from the interviews as three quarters of our participants mentioned it, equally distributed among American and Swiss researchers. Numerous scholars emphasized how informed consent is at the foundation of appropriate research practices. P2, a Swiss psychologist, noted:

But of course it's pretty clear to me informed consent is very important and it’s crucial that people know what it is what kind of data is collected and when they would have the possibility of saying no and so on. I think that’s pretty standard for any type of data. (…) I mean it all goes down to informed consent.

For a few of our participants, in the era of Big Data, it becomes not really a matter of consent but a matter of awareness. Since research with Big Data could theoretically be performed without the knowledge of the participant, research subjects at least have to be made aware that they are part of a research project as claimed by P38 a Swiss sociologist who said:

I think that everything comes down to the awareness of the subject about what is collected about them. I mean, we have collected data for ages, right? And I mean, before it was using pen and paper questionnaires, phone interviews or…there’s been data collection about private life of people for, I mean, since social science exists. So, I think the only difference now is the awareness.

Another practice that was considered fundamental by our participants was the right of participants to withdraw from a research study that, in turn, was translated in giving the participants more control over their data in the context of Big Data research. For example, while describing their study with social media, a Swiss sociologist (P38) explained that”the condition was that everybody who participated was actually able to look at his own data and decide to drop from the survey any time”. Another Swiss sociologist (P37), when describing a study design in which they asked participants to install an add-on on their browser to collect data on their Facebook interactions, underlined the importance of giving participants control over their data and to teach them how to manage them, in order to create a trust based exchange between them and the investigators:

And there you'd have to be sure that people…it's not just anonymizing them, people also need to have a control over their data, that's kind of very important because you need kind of an established trust between the research and its subjects as it were. So they would have the opportunity of uninstall the…if they're willing to take part, that's kind of the first step, and they would need to download that add-on and they'd also be instructed on how to uninstall the add-on at any point in time. They'd be also instructed on how to pause the gathering of their data at any point in time and then again also delete data that well…at first I thought it was a great study now I'm not so sure about, I want to delete everything I've ever collected.

The same researcher suggested to create regulations that ensure ownership of research data to participants in order to allow them to have actual power over their participation past the point of initial consent.

And legal parameters then should be constructed as such that it has to be transparent, that it guards the rights of the individual (…) in terms of having ownership of their data. Particularly if it's private data they agree to give away. And they become part of a research process that only ends where their say. And they can always withdraw the data at any point in time and not just at the beginning with agreeing or not agreeing to taking part in that. But also at different other points in time. So that i think the…you have to include them more throughout your research process. Which is more of a hassle, costs more money and more time, but in the end you kind of. . . .it makes it more transparent and perhaps it makes it more interesting for them as well and that would have kind of beneficial effects for the larger public I suppose.

In addition, transparency of motives and practices was also considered a fundamental principle for digital research. For instance, transparency was seen as a way for research participants to be fully informed about the research procedures and methods used by investigators. According to a few participants transparency is key to guarantee people’s trust the research system and to minimize their worry and reservations about participating in research studies. On this P14, an American psychologist, noted:

I think we need to have greater transparency and more. . . . You know our system, we have in the United States is that…well not a crisis, the problem that we face in the United States which you also face I'm sure, is that…you know, people have to believe that this is good stuff to do (participating in a study). And if they don't believe that this is good stuff to do then it's a problem. And so. . . .so I think that that. . . .and I think that the consent process is part of it but I think that the other part of it is that the investigators and the researchers, the investigators and the institutions, you know, need to be more transparent and more accountable and make the case that this is something worth doing and that they're being responsible about it.

A Swiss sociologist, P38, who described how they implemented transparency in their research project by giving control to participants over the data they were collecting on them, highlighted that the fear individuals might have towards digital and Big Data research might come from lack of information and understanding about what data investigators are collecting on them and how they are using it. In this sense transparency of practices not only ensures that more individuals trust the research systems, but it will also assist them in making a truly informed decision about their participation in a study.

And if I remember correctly the conditions were: transparency, so every subject had to have access to the full data that we were collecting. They had also the possibility to erase everything if they wanted to and to drop from the campaign. I guess it's about transparency. (…) So, I think this is key, so you need to be transparent about what kind of data you collect and why and maybe what will happen to the data. Because people are afraid of things they don't understand so the better they understand what's happening the more they would be actually. . . . not only they will be willing to participate but also the more they will put the line in the right place. So, this I agree, this I don't agree. But the less you understand the further away you put the line and you just want to be on the safe side. So, the better they understand the better they can draw the line at the right place, and say ok: this is not your business, this I'm willing to share with you.

In addition, one of our participants considered transparency to be an important value also between scholars from different research teams. According to this participant, open and transparent communication and exchange between research would help implement appropriate ethical norms for digital research. They shared:

But I think part of it is just having more transparency among researchers themselves. I think you need to have like more discussions like: here's what I'm doing…here's what I'm doing…just more sharing in general, I think, and more discussion. (…) People being more transparent on how they're doing their work would just create more norms around it. Because I think in many cases people don't know what other people have been doing. And that's part of the issues that, you know, it's like how do I apply these abstract standards to this case, I mean that can be though. But if you know what everybody is doing it makes a little bit easier. (P3-US, Sociologist)

On the other hand, however, a sociologist from Switzerland (P37), noted that the drive towards research transparency might become problematic for ensuring the anonymity of research participants as more information you share about research practices and methods the more possibilities of backtracking and re-identifying the participants to the study.

It’s problematic also because modern social science, or science anyway, has a strong and very good drive towards transparency. But transparency also means, that the more we become transparent the less we can guarantee anonymity (…) If you say: "well, we did a crawl study", people will ask "well, where are you starting, what are your seeds for the crawler?". And it's important to, you know, to be transparent in that respect.

#### Privacy and anonymity

Respect for the privacy of research participants, and protection from possible identification, usually achieved through anonymization of data, were the second most mentioned standards to be considered while conducting Big Data research. P33, a Swiss sociologist, underlined how “If ever, then privacy has…like it’s never been more important than now”, since information about individuals is becoming increasingly available thanks to digital technologies, and how institutions now have a responsibility to ensure that such privacy is respected. A Swiss data scientist, P29, described the privacy aspect embedded in their research with social media and how their team is constantly developing strategies to ensure anonymity of research subjects. They told:

Yeah, there is a privacy aspect of course, that's the main concern, that you basically…if you’re able to reconstruct like the name of the person and then the age of the person, the address of the person, of course you can link it then to the partner of the person, right? If she or he has, they're sharing the same address. And then you can easily create the story out of that, right? And then this could be an issue but…again, like we try to reapply some kind of anonymization techniques. We have some people working mostly on that. There is a postdoc in our group who is working on anonymization techniques.

Similarly, an American researcher, P6 Sociologist, underlined how it should become a routine practice for every research project to consider and implement practices to protect human participants from possible re-identification:

In the social science world people have to be at least sensitive to the fact that they could be collecting data that allows for the deductive identification of individuals. And that probably…that should be a key focus of every proposal of how do you protect against that.

### Challenges introduced by Big Data to research ethics and ethical principles

A consistent number of our researcher, on the other hand, recognized how Big Data research and methods are introducing numerous challenges related to the principles and practices they consider fundamental for ethical research and reflected upon the limits of the traditional ethical principles.

When discussing informed consent, participants noted that that it might not be the main standard to refer to when creating ethical frameworks for research practices as it cannot be ensured anymore in much digital research. For instance, P14, an American psychologist noted:

I think that that the kind of informed consent that we, you know, when we sign on to Facebook or Reddit or Twitter or whatever, you know, people have no idea of what that means and they don't have any idea of what they're agreeing to. And so, you know the idea that that can bear the entire weight of all this research is, I think…I think notification is really important, you can ask for consent but the idea that that can bear the whole weight for allowing people to do whatever/ researchers to do whatever they want, I think it's misguided.

Similarly, P18, an American scholar with a background in data science, felt that although there is still a place for informed consent in the digital era, this practice should be appropriately revisited and reconsidered as it cannot be applied anymore in the stricter sense, for instance when analyzing aggregated databases where personal identifiers are removed and it would be impossible to trace back the individual to ask them for consent. Data aggregation is the process of gathering data from multiple sources and presenting it in a summarized format. Through the process of data aggregation, data can be stripped from personal identifiers thus ensuring anonymization of the dataset and analyzing aggregate data should, theoretically not reveal personal information about the user. The participant shared:

Certainly, I think there is [space for informed consent in digital research]. And like I said I think we should require people to have informed consent about their data being used in aggregate analysis. And I think right now we do not have informed consent. (…) So, I think again, under the strictest interpretation even to consent to have one’s data involved in an aggregate analysis should involve that. But I don't know, short of that, what would be an acceptable tradeoff or level of treatment. Whether simply aggregating the analysis is good enough and if so what level of aggregation is necessary.

As for consent, many of our participants while recognizing the importance of privacy and anonymity, also reflected on some of the challenges that Big Data and digitalization of research are creating for these research standards. First, a few respondents highlighted how in digital research the risk of identification of participants is quite high as anonymized datasets could almost always be de-anonymized, especially if data is not adequately secured. On this, P1, an American sociologist explained:

I understand and recognize that there are limits to anonymization. And that under certain circumstances almost every anonymized dataset can be de-anonymized. That's what the research that shows us. I mean sometimes that requires significant effort and then you ask yourself would someone really invest like, you know, supercomputers to solve this problem to de-anonymize…

A Swiss sociologist (P38) described how anonymization practices towards the protection of the privacy of the research participant could, on the other hand, diminish the value of the data for research as anonymization would destroy some of the information the researcher is actually interested in.

You know, we cannot do much about it. So… there is a tendency now to anonymize the data but basically ehm…anonymization means destruction of information in the data. And sometimes the information that is destroyed is really the information we need…

Moreover, it was also claimed how digital practices in research are currently blurring the line between private and public spaces creating additional challenges for the protection of the privacy of the research participant and practices of informed consent. A few of our researchers highlighted how research subjects might have an expectation of privacy even in public digital spaces such as social media and public records. In this context, an American sociologist, P9, noted how participants could have a problem in allowing researchers to link together publicly available datasets as they would prefer information stemming from this linkage to remain private:

P9USR: Well because the question is…even if you have no expectation of privacy in your Twitter account, you know Twitter is public. And even if you have no expectation of privacy in terms of whether you voted or not, I don't know, in Italy maybe it's a public record whether if you show up at the pool or not. Right? I can go to the city government and see who voted in the last elections right? (…) So…who voted is listed or what political party they're member of is listed, is public information. But you might have expectation of privacy when it comes to linking those data. So even though you don't expect privacy in Twitter and you don't expect privacy in your voting records, maybe you don't like it when someone links those things together.

In addition, a sociologist, P19 from the US, noted how even with just linking information of some publicly available data, research subjects could be easily identified.

However, when one goes to the trouble of linking up some of the aspects of these publicly available sets it may make some individuals identifiable in a way that they haven't been before. Even though one is purely using publicly available data. So, you might say that it kind of falls into an intermediate zone. And raises practical and ethical questions on protection when working with publicly available data. I don't know how many other people you have interviewed who are working in this particular grey zone.

Two of our participants while describing personal strategies to handle matters of expectation of privacy and consent, discussed the increased blur between private and public spaces and how it is becoming increasingly contextual to adequately handle matters of privacy on social media.

P2USR: So, for example when I study journalists, I assume that their Tweets are public data just because Twitter is the main platform for journalists to kind of present their public and professional accomplishments and so I feel fine kind of using their tweets, like in the context of my research. I will say the same thing, about Facebook data for example. So, some of the journalists kind of… that I interviewed are… are not on Facebook anymore, but at the time we became friends on Facebook and there were postings and I… I wouldn't feel as comfortable, I wouldn't use their Facebook data. I just think that somehow besides the norms of the Facebook platform is that it's more private data, from…especially when it's not a public page so… But it's like… it's fuzzy.

### Responsibility and ethical reflection in research

Due to the challenges introduced by digital methods, some of our participants elaborated on their opinions regarding the role of ethical reflection and their responsibility in addressing such challenges in order to ensure the protection of research participants.

Among them, some researches emphasized the importance for investigators to apply ethical standards to appropriately perform their research projects. However, a couple of them recognized how not all researchers might have the background and expertise to acknowledge the ethical issues stemming from their research projects or to be adequately familiar with ethical frameworks. On this, P12, an American sociologist, highlighted the importance of education in ethics for research practitioners:

I also want to re-emphasize that I think that as researchers in this field we need to have training in ethics because a lot of the work that we're doing (pause) you know can be on the border of infringing on people’s privacy.

In addition, self-reflection, ethical interrogation and evaluation about the appropriateness of certain research practices was a theme that emerged quite often during our interviews. For an American psychologist, P4, concerned about issues of consent in digital research, it is paramount that investigator begin to interrogate themselves upon what type of analysis would be ethically appropriate without explicit consent of participants.

And it is interesting by the way around Big Data because in many cases those data were generated by people who didn't sign any consent form. And they have their data used for research. Even (for the) secondary analysis of our own data the question is: what can you do without consent?

Similarly, P26, a sociologist from Switzerland, reflected upon the difficulties that researchers might encounter in evaluating what type of data investigators can consider unproblematic to collect and analyze even in digital public spaces, like social media:

Even though again, it's often not as clear cut, but I think if people make information public that is slightly different from when you are posting privately within a network and assume that the only people really seeing that are your friends. I see that this has its own limits as well because certain things…well A: something like a profile image I think is always by default public on Facebook…so… there you don't really have a choice to post it privately. I guess your only choice is not to change it ever. And then the other thing is that…I know it because I study (…) internet skills, I know a lot of people are not very skilled. So, there are a lot of instances where people don't realize they're posting publicly. So even if something is public you can't assume people had meant it to be public.

Moreover, reflection and evaluation of the intent behind a research study was considered important by P31, a Swiss data scientist, for ethical research in Big Data. The researcher recognized that this is difficult to put into practice as investigators with ill intent might lie about their motivations and you could have negative consequences even with the noblest of intents.

I find it really difficult to answer that. I would say, the first thing that comes to my mind is the evaluation of intent… rather than other technicality. And I think that's a lacking point. But also the reason why I don't give that answer immediately is like…intent is really difficult to probe… and it's probably for some people quite easy to know what is the accepted intent. And then I can of course give you a story that is quite acceptable to you. And also with good intent you can do evil things. So, it's difficult but I would say that discussion about the intent is very important. So that would be maybe for me a minimal requirement. At least in the discussions.

In this context, some scholars also discussed their perception regarding responsibility of protecting research participants in digital studies and the role investigators play in overcoming ethical issues.

For a few of them it was clear that the responsibility of protecting the data subjects should fall on the investigators themselves. For instance, an American scholar, P22 sociologist, while discussing the importance of creating an ethical framework for digital research that uses publicly available data of citizens shared:

So, I do think (the responsibility) it's on researchers (…) and I get frustrated sometimes when people say "well it's not up to us, if they post it there then it's public". It's like well it is up to us, it's literally our job, we do it all day, try to decide, you know, what people want known about them and what people don't. So, we should apply those same metrics here.

However, other researchers also pointed out how the introduction of digital technologies and digital methods for behavioral research is currently shifting the perceived responsibility scholars have. P16, an American sociologist, shared some concerns regarding the use of sensor devices for behavioral research and reflected on how much responsibility they, as investigators, have in assuring data protection of their research subjects since the data they work with is owned by the company that provided the device for data collection:

There's still seems to be this question about…whether. . . .what the Fitbit corporation is doing with those data and whether we as researchers should be concerned about that. We're asking people to wear Fitbits for a study. Or whether that's just a separate issue. And I don't know what the answer to that is, I just know that it seems like the type of question that it's going to come up over and over and over again.

One a similar note, P14, an American psychologist, noted that while researchers actually have a responsibility of preventing harm that might derive from data research, it should be a responsibility in part shared with data holders. They claimed:

Do I think that the holders of data have a responsibility to try to you know, try to prevent misuse of data? Yeah, I think they probably do. (…) I think there is a notion of stewardship there. Then I think that investigators also have an independent obligation to make sure to think about the data they're analyzing and trying to get and think about what they're using it for. So not to use data in order to harm other people or those kinds of things.

Finally, a few participants hinted at the fact that research ethics boards like Institutional Review Boards (IRBs) and Ethics Committees (ECs) should play a bigger role of responsibility in ensuring that investigators actually perform their research ethically. For instance, P16, an American sociologist, complained that IRBs do not provide adequate follow-up to researchers to ensure that they are appropriately following the approved research protocols.

There does seem to be kind of a big gap even in the existing system. Which is that a researcher proposes a project, the IRB hopefully works with the researcher and the project gets approved and there's very little follow-up and very little support for sort of making sure that the things that are laid out at the IRB actually in the proposal and the project protocol actually happen. And not that I don't believe that most researchers have good intensions to follow the rules and all of that but there are so many of kind of different projects and different pressures that things can slip by and there's… there's nobody.

## Discussion

As Big Data methodologies are becoming widespread in research, it is important to reach international consensus on whether and how traditional principles for research ethics, such as the ones described in the Belmont Report, are still relevant for the new ethical questions introduced by Big Data and internet research [22, 23]. Our study offers a relevant contribution to this debate as it investigated the methodological strategies and code of ethics researchers from different jurisdictions—Swiss and American investigators—apply in their Big Data research projects. It is interesting to notice how, despite regional difference, participants shared very similar ethical priorities. This might be due to the international nature of academic research, where scholars share similar codes of ethics and apply similar strategies for the protection of research participants.

Our results point out that in their code of conduct, researchers mainly referred to the traditional ethical principles enshrined in the Belmont report and the Declaration of Helsinki, like respect for persons in the practice of informed consent, beneficence, minimization of harm through protection of privacy and anonymization, and justice. This finding shows that such principles are still considered relevant in behavioral sciences to address the ethical issues of Big Data research, despite the critique of some that rules designed for medical research cannot be applied in sociological research [36]. Even before the advent of Big Data, the practical implementation of the Belmont Report principles has never been an easy endeavor as they were originally conceived to be flexible to accommodate a wide range of different research settings and methods. However it has been argued that exactly this flexibility makes them the perfect framework in which investigators can “clarify trade-offs, suggest improvements to research designs, and enable researchers to explain their reasoning to each other and the public” in digital behavioral research [2].

Our study shows how scholars still place great importance on the practice of informed consent. They considered crucial that participants are appropriately notified of their research participation, are adequately informed about at least some of the details and procedures of the study, and are given the possibility to withdraw at any point in time. A recent study, however, has highlighted that there is currently no consensus among investigators on how to collect meaningful informed consent among participants in digital research [37]. Similarly, a few researchers from our study recognized that consent, although preferable in theory, might not be the most adequate practice to refer to when designing ethical frameworks. In the era of Big Data behavioral research, informed consent becomes an extremely complex practice that is intrinsically dependent on the context of the study and the type of Big Data used. For instance, in certain behavioral studies that analyze track data from devices related to a limited number of participants, it would be feasible to ask for consent prior to beginning of the study. However, recombination and reanalysis of the data, possibly across ecosystems far removed from the original source of the data, makes it very difficult to fully inform participants about the range of uses to which their data would be put through, the type of information that could emerge from the analysis of the data, and the unforeseeable harms that the disclosure of such information could cause [38]. In online studies and internet-mediated research, consent often amounts to an agreement to unread terms of service or a vague privacy policy provided by digital platforms [18]. Sometimes valid informed consent is not even required by official guidelines when the analyzed data can be considered ‘in the public domain’ [39], leaving participants unaware that research is performed on their data. It has been argued however that researchers should not just assume that public information is freely accessible for collection and research just because it is public. Researchers should take into consideration what the subject might have intended or desired regarding the possibility for their data to be used for research purposes [40]. At the same level, we can also argue that even when information is harvested with consent, the subject might a) not wish for their data to be analyzed or reused outside of the purview of the original research purpose and b) fail to understand what is the extent of the information that the analysis of the dataset might reveal about them.

Matzner and Ochs argue that practices of informed consent “are widely accepted since they cohere with notions of the individual that we have been trained to adopt for several centuries” [41], however they also emphasize how such notions are being altered and challenged by the openness and transience of data-analytics that prevent us from continuing to consider the subject and the researcher within a self-contained dynamic. Since *respect for persons*, in the form of informed consent, is just one of the principles that needs to be balanced when considering research ethics [42], it becomes of outmost importance to find the right balance between the perceived necessity of still ensuring consent from participants and the reality that such consent is sometimes impossible to obtain properly. Salganik [2], for instance, suggests that in the context of digital behavioral research rather than “informed consent for everything”, researchers should follow a more complex rule: “some form of consent for most things”. This means that, assuming informed consent is required, it should be evaluated on a case by case basis whether consent is a) practically feasible and b) actually necessary. This practice might however leave too much space to the discretion of the investigator who might not have the skills to appropriately evaluate the ethical facets of their research projects [43].

Next to consent, participants from our study also argued in favor of ensuring more control to participants over their own data. In the past years, in fact, it has been argued that individuals often lack the control to manage, protect and delete their data [20, 28]. Strategies of dynamic consent could be considered a potential tool to address ethical issues related to consent in Big Data behavioral research. Dynamic consent, a model where online tools are developed to have individuals engage in decisions about how their personal information should be used and which allows them some degree of control over the use of their data, are currently mainly developed for biomedical Big Data research [44, 45]. Additional research could be performed to investigate if such models can be translated and applied also for behavioral digital research.

Strictly linked to consent is the matter of privacy. Many researchers underlined the importance of respecting the privacy and anonymity of research participants to protect them from possible harm. At the same time, they also recognized the many challenges related to such practice. They highlighted the difficulty of ensuring complete anonymity of the data and prevent re-identification of participants in Big Data research, especially since high level of anonymization could cause the loss of essential information for the research project. The appropriate trade-off between ensuring maximum anonymization for participants while maintaining quality of the dataset is still hotly debated [12]. Growing research in data science strives towards developing data models to ensure maximum protection for participants [46]. On the other hand, our participants also referred to the current debate surrounding the private nature of personal data as opposed to publicly available data and how Big Data and digital technologies are blurring the line between private and public spheres. Some respondents expressed concern or reservation towards the analysis of publicly available data–especially without informed consent–as it could still be considered an infringement of the privacy of research participants and also cause them harm. This shows how researchers are well aware of the problems of considering privacy a binary concept (private vs public data) and that they are also willing to reflect upon strategies to protect the identity of participants even when handling publicly available data. According to Zook et al. [47], breaches of privacy are the main means by which Big Data can do harm as it might reveal sensitive information about people. Besides the already mentioned “Tagging Banksy” project [17], another distressing example is what happened in 2013, after the New York City Taxi & Limousine Commission released an anonymized dataset of 173 million individual cab rides–including the pickup and drop-off times, locations, fare and tip amount. Many researchers who freely accessed this database showed how easy it was to elaborate the dataset so that it revealed private information about the taxi-drivers, such as their religious belief, average income and even an estimation of their home address [48]. It becomes therefore increasingly crucial that investigators in the behavioral sciences recognize how privacy is contextual, situational and changes over time as it depends on multiple factors such as the context in which the data were created and obtained, and the expectations of those whose data is used [2, 47, 49, 50]. For instance, as reported by one of our respondents, users might not have expectations of privacy on some publicly available information when taken singularly or separately–e.g. social media and voter data, but they might have privacy concerns on the information that the linkage of this data might reveal–e.g. who they voted for. This difficulty, if not impossibility, of defining a widespread single norm or rule for protecting privacy, shows again the intrinsic context dependency of Big Data studies, and highlights how researchers are increasingly called to critically evaluate their decisions on a case by case basis rather than by blindingly applying a common rule.

As new methods of data collection and analysis in behavioral sciences create controversy and appropriately balancing and evaluating ethical principles is becoming a source of difficult decisions for researchers [2], our participants underlined the importance of ethical reflection and education towards the appropriate development of research projects. They also recognized how investigators are called to critically reflect about the design of their studies and the consequences they might have for research participants [51]. However, as claimed by one of our participants, not all researchers, especially those coming from more technical disciplines like data science, might have the expertise and tools to proactively think about ethical issues when designing a research project [22] and might need additional guidance. We therefore argue that education in ethics, exchange and dialogue between research teams and scholars from different disciplines must be implemented. As suggested by Zook et al. [47] discussion and debate of ethical issues are an essential part of establishing a community of ethical practitioners and integrating ethical reflection into coursework and training can enable a bigger number of scholars to raise appropriate ethical questions when reviewing or developing a project.

Within the current discussion, we have seen how context-dependency, although never spelled out explicitly by our participants, becomes a major theme in the debate over ethical practices in Big Data studies. Our results have in fact highlighted that a one-size fits all approach to research ethics, or a definite overarching set of norms or rules to protect research participants, is not opportune to appropriately handle the multifaceted ethical issues of Big Data. The context-dependent nature of some of the ethical challenges of Big Data studies, such as consent and privacy, might require a higher level of flexibility together with a more situational and dialogic approach to research ethics [23]. For instance, the Association of Internet Researchers (AoIR) in the development of their Ethical Guidelines for Internet research agrees that the adequate process approach for ethical internet research is one that is reflective and dialogical “as it begins with reflection on own research practices and associated risks and is continuously discussed against the accumulated experience and ethical reflections of researchers in the field and existing studies carried out” [52]. As a consequence we argue that applying context specific assessments increases the chances of solving ethical issues and appropriately protecting research participants [53]. Many authors in the field are thus promoting methodological approaches that focus on contextually-driven decision-making for Big Data research. Zimmer, for example, suggests the application of contextual integrity’s decision heuristic on different research studies to appropriately assess the ethical impact of the study on the privacy of its participants and consequently overcome the conceptual gaps left by the Belmont Report for Big Data research ethics [50]. Similarly, Steinmann et al. [53] provide an heuristic tool in the form of a “privacy matrix” to assist researchers in the contextual assessment of their research projects.

But what should drive investigators’ ethical reflection and decision making? Despite the multifaceted challenges introduced by Big Data and digital research, we argue that the principles stated in the Belmont Report can still be considered a valuable guidance for academic investigators. As argued by Rothstein [28], we believe Big Data exceptionalism is no viable option and new challenges should not serve as a catalyst for abandoning foundational principles of research ethics. This is in line with the current best practices suggested by institutional bodies like the American Psychological Association (APA), that claim that the core ethical principles set by the Belmont report should be expanded to address the risks and benefits of today’s data [6]. Numerous research groups are striving towards the design of ethical frameworks in Big Data research that stay true to the foundational principles of research ethics, but at the same time accommodate the needs and changes introduced by Big Data methods. Steinmann et al. [53], for instance, suggest to consider five principles (non-maleficence, beneficence, justice, autonomy, and trust) as a well-defined pluralism of values that, by having clear and direct utility in designating practical strategies for protecting privacy, should guide researchers in the evaluation of their research projects. Xafis et al. [38], in the development of an ethical framework for Biomedical Big Data research, provide a set of 16 values relevant for many Big Data uses divided in substantive values (such as justice, public benefit, solidarity or minimization of harm) and procedural values (accountability, consistency, transparency and trustworthiness) that should be used by investigators to identify and solve ethical issues within their research project. Vitak et al. [22] recommend the implementation of the principle of transparency, intended as a flexible principle that finds application in different ethical components related both to intent of research (what you are doing with data and why) and practice (how you’re getting the data–informed consent (disclosing purpose and potential use) and how you are processing the data–data anonymity). Also, according to some of our participants, enhancement of transparency in research practices would be positive on different levels. First, it would assist participants in trusting the research system and minimize their worry about participating in research studies; in addition, enhanced transparency between research teams would assist in building up the knowledge to face the ethical issues that emerge in heterogeneous research projects. Although the principle of transparency is becoming increasingly embedded in research practices as something highly recommended, there is still some uncertainty regarding how this principle would actually translate in practice, in order to overcome challenges posed to ethical practices like consent. At the moment much of the debate on transparency mainly focuses on the implementation of algorithmic transparency with Big Data [54], more research should focus on how put research transparency in practice

Finally, a very relevant theme that our participants reflected upon, that it is rarely addressed by the current literature on Big Data studies, was the topic of responsibility. Some of our respondents in fact interrogated themselves whether the introduction of digital technologies and methods implies a shift of responsibility in protecting research participants. Although all those who discussed responsibility admitted that at least part of it should definitely fall on investigators themselves, some pointed that also other actors involved in Big Data research could share some of this responsibility such as data holders, data owners–in case of the use of corporate data. Digital research has in fact changed the traditional research subject/investigator dynamic [18] by introducing other factors/actors in the process (social media platforms, private firms etc.) and therefore raises ethical challenges for which researchers do not always have the necessary skills to either anticipate or face [25, 43]. To the best of our knowledge, it seems that this aspect of responsibility has not yet entered the ethics debate. This might be due to the practical difficulties that such a debate would necessarily imply such as communication, coordination and compromise between stakeholders with very different goals and interests at stake [55, 56]. However, our results show that there are relevant questions and issues that should be further addressed such as: who should bear the responsibility of protecting the research subject in Big Data studies? How much should data owners, data holders, ethics committees and even users be involved in sharing such responsibility? We believe that academic investigators should not bear all the responsibility of the ethical design of research projects alone, or singularly confront themselves with the ethical implications of digital research [57]. At the moment, models of consultancy between ethics committees and researchers are advocated to assist investigators foresee ethical issues [25, 43]. These models, together with the implementation of sustainable and transparent collaboration/partnership with data holders and owners [58], could assist the creation of appropriate paradigms of shared responsibility that could definitely play a significant role in the development of ethically sound research projects.

### Limitations

First, since our respondents were mainly from the fields of psychology and sociology, the study might have overlooked the perspectives of other relevant fields for human subject research that make use of Big Data methodologies (e.g., medicine, nursing sciences, geography, urban planning, computer science, linguistics, etc.). In addition, the findings of this study are based on a small sample of researchers from only two countries that share similar ethical norms and values. For these reasons, the findings from this analysis are not generalizable globally. Future research that takes into account additional disciplines and different countries might contribute to delivering a more comprehensive understanding of the opinions and attitudes of researchers. Finally, a limitation must be acknowledged regarding the definition of Big Data used for this study. Using the term Big Data as an umbrella term prevented us from undertaking a more nuanced analysis of the different types of data used by our participants and their specific characteristics (for instance the different ethical challenges posed by online social media data as compared to sensor data obtained with the consent of the participants). In our discussion we referred to the contextual dependency of the ethical issues of Big Data and the necessity of a continuous ethical reflection that assesses the specific nuances of the different types of Big Data in heterogeneous research projects. However we already recognized the risks of conceptualizing Big Data as a broad overarching concept [33]. As a consequence, we believe that future research on Big Data ethics will benefit from a deconstruction of the term into its different constituents in order to provide a more nuanced analysis of the topic.

## Conclusion

This study investigated the code of ethics and the research strategies that researchers apply when performing Big Data research in the behavioral sciences and it also illustrates some of the challenges scholars encounter in practically applying ethical principles and practices. Our results point out how researchers find the traditional principles of the Belmont Report to be a suitable guide to perform ethical data research. At the same time, they also recognized how Big Data methods and practices are increasingly challenging such principles. Consent and protection of privacy were considered still paramount practices in research. However, they were also considered the most challenged practices since digitalization of research has blurred the boundary between “public and private” and made obtaining consent from participants impossible in certain cases.

Based the results and discussion of our study, we suggest three key items that future research and policymaking should focus on:

- Development of research ethics frameworks that stay true to the principles of the Belmont Report but also accommodate the context dependent nature of the ethical issues of Big Data research;
- Implementation of education in ethical reasoning and training in ethics for investigators from diversified curricula: from social science and psychology to more technical fields such as data science and informatics;
- Design of models of consultancy and shared responsibility between the different stakeholders involved in the research endeavor (e.g. investigators, data owners and review boards) in order to enhance protection of research participants.

## Supporting information

S1 File Interview guide. Semi structured interview guide that illustrates the main questions and themes that the researchers asked to the participants (questions relevant for this study are highlighted in yellow).
(DOCX)
